# Myths and Misconceptions of Breast Cancer in the Pakistani Population

**DOI:** 10.7759/cureus.40086

**Published:** 2023-06-07

**Authors:** Sulhera Khan, Sumeen Jalees, Zohra Jabeen, Marium Khan, Rafia H Qadri, Haneya Adnan, Bareerah S Khan, Amir H Khan

**Affiliations:** 1 Dermatology, Dow University of Health Sciences, Civil Hospital Karachi, Karachi, PAK; 2 Internal Medicine, Jinnah Postgraduate Medical Centre, Karachi, PAK; 3 Internal Medicine, Dow University of Health Sciences, Civil Hospital Karachi, Karachi, PAK; 4 Community Medicine, Karachi Medical and Dental College, Karachi, PAK

**Keywords:** breast cancer screening, prevention, pakistani population, muslim population, misinformation, misconception, myths, breast cancer

## Abstract

Background

Breast cancer is one of the most prevalent types of cancer in the female population. The cultural diversity, religious beliefs, myths, and misinformation regarding the disease contribute to diagnostic delays and enhanced burden on the healthcare system. This study aimed to ascertain the extent of knowledge and prevalence of erroneous beliefs and misconceptions regarding breast cancer among Pakistani women belonging to diverse socioeconomic and educational backgrounds.

Methodology

This cross-sectional study was performed in a tertiary care hospital in Karachi, Pakistan. A total of 350 women were enrolled in the study as a representative female population, and 300 participants were included who met the inclusion criteria. Participants were conveniently interviewed using a pre-piloted questionnaire designed to assess the prevalent myths and misconceptions about breast cancer. The data were analyzed by SPSS version 23 (IBM Corp., Armonk, NY, USA) using descriptive statistics.

Results

The study findings point to a significant prevalence of erroneous beliefs and a lack of accurate information on breast cancer. The mean age of the participants was 20.8 ± 10.4 years. The majority of the participants belonged to a middle socioeconomic status (70%) and were undergraduates (61.4%). The participants’ friends and family members were the most frequent sources of information regarding breast cancer. The most common myth was “breast-feeding offers immunity to breast cancer completely” (76.6%), followed by “breast cancer spreads after biopsy” (63.8%). Participants also believed that breast tissue biopsy can lead to the spread of cancer (63.4%) and that faith healers and alternative medicine can cure breast cancer (47.5%). One-third (33.3%) of the participants considered all lumps to be breast cancer; however, approximately half (41.6%) of the participants thought that only painful lumps were associated with breast cancer. A significant number of participants believed breast cancer to be a result of God’s curse (31.4%) or evil eye (38.7%).

Conclusions

The findings suggest a critical need for community-based breast health education initiatives that take into account Pakistani women’s distinctive cultural and societal attitudes and work to dispel common misconceptions about the condition.

## Introduction

The annual burden of breast cancer worldwide is 12.5% of new cancer cases. Globally, breast cancer is the most common cause of cancer and cancer-related deaths [1]. It has been estimated that more than one million women are diagnosed with breast cancer worldwide annually, with approximately 400,000 women dying secondary to this illness globally [2].

Cancer is more common in the developed world but the prevalence of cancer is increasing in low-to-middle-income countries (LMICs) like Pakistan [3]. It has been estimated that approximately 70% of breast cancer-related deaths occur in LMICs [4]. This can be attributed to a delay in diagnosis due to various myths, misconceptions, and cultural beliefs, as well as due to a deficiency in diagnostic modalities [5]. It can also be linked to the deficiency of healthcare policies and guidelines for early detection and advancements in treatment modalities and interventions [6]. The prevalence of breast cancer is the highest in Asian countries like India and Pakistan [7]. It has been reported that approximately 178,388 new breast cancer cases were registered in Pakistan in 2020 [8].

Myths and misconceptions contribute to the increased breast cancer burden in Pakistan. The misinformation regarding the clinical presentation, diagnosis, and management of breast cancer hinders early diagnosis, creates a negative doctor-patient relationship, and hinders the approach toward management. Therefore, misinformation, myths, and misconceptions have been labeled as global risks [9]. Cultural beliefs also contribute to the increased myths in society. The misinformation allows the patients to utilize harmful interventions in the hope of seeking a cure [2]. This might result in disastrous outcomes and increased morbidity.

In this scenario, considering the high prevalence of breast cancer misinformation in our population combined with a low literacy rate, it is imperative to eliminate the factors contributing to breast cancer misinformation to enhance the dissemination of correct information regarding breast cancer diagnosis and management [2]. The commonly used techniques for the diagnosis of breast cancer include monthly breast self-examination which is seen to decrease the mortality of breast cancer by 18% [4]. This is supported by the fact that early detection of smaller tumors minimizes the risk of metastasis and is a major factor contributing to overall improved mortality [10]. It has been estimated that the majority of females on their first presentation to breast cancer clinics have stage III or IV disease (69.9%) [11]. The delayed presentation also leads to an increased financial burden on the country.

We conducted a study in a tertiary care hospital in Karachi, Pakistan, on healthy females to determine the prevalence of myths and misconceptions about breast cancer in our population. Identifying the popular myths can help in developing awareness programs and newer guidelines to decrease the burden of misinformation contributing to diagnostic delay and management. We also evaluated the relationship between literacy and education status and its association with the prevalence of myths and misconceptions to provide a perspicacious approach to enhancing the development of healthcare policies and education.

## Materials and methods

A total of 350 participants were enrolled in a cross-sectional study conducted at the outpatient department of a tertiary care hospital in Karachi, Pakistan. These participants were selected based on a study conducted at the Aga Khan University Hospital Karachi and Karachi Institute of Radiation and Nuclear Medicine (KIRAN) cancer hospital in 2020 on the patient delay in breast cancer diagnosis in two hospitals in Karachi, Pakistan [10]. Institutional Review Board approval was obtained from Jinnah Sindh Medical University (approval number: JSMU/ IRB/ 2016/ -4.8).

The sample size was calculated using the Open Epi sample size calculator. A random convenience sampling technique was used to select the participants. All patients were interviewed face to face and written consent was taken from all participants. The interview was conducted by the researchers themselves. The purpose of the research and the procedure of data collection were explained to the participants. The patients were asked to fill out a preformed performa designed after a thorough literature search and review. Information was obtained regarding the myths and misconceptions and cultural beliefs about breast cancer, and their approach toward diagnosis and treatment was recorded. The self-designed questionnaire was translated into Urdu, the national language of Pakistan. Translators were arranged for participants who were unable to read both Urdu and English languages. The inclusion criteria included all healthy females aged 20-70 years with or without a history of breast cancer. All participants denying consent were excluded from the study.

The data were fed in SPSS version 23 (IBM Corp., Armonk, NY, USA) and analyzed using descriptive statistics. The demographics of the participants were recorded. The socioeconomic status was divided into three categories depending upon the income of the participants such as lower class with an income of <50,000 Pakistani Rupees (PKR), middle class with an income of 50,000-100,000 PKR, and high class with an income of >100,000. The education status was divided into four categories such as no education (unable to read and write), primary education (undergraduate), secondary education (graduate), and tertiary education (postgraduate). All questions for myths and misconceptions were scored (one point for a correct response and zero for an incorrect or not sure response). The determinant of the correct responses was based on the review of current guidelines and literature. The categorical variables were evaluated as frequencies and means. The level of correlation between education status and the prevalence of misconceptions was determined using the chi-square test. A p-value of 0.05 was considered significant for all tests.

## Results

A total of 350 participants were enrolled in the study; however, 50 participants were excluded due to failure to meet the inclusion criteria. Twenty-five participants were excluded as they had no knowledge or awareness regarding breast cancer. Sixteen participants were excluded because they failed to sign the consent form, and nine participants were excluded because of incomplete data.

The demographic characteristics of the participants are summarized in Table 1. The mean age was 20.8 ± 10.4. The majority of the participants belonged to a middle socioeconomic status (70%) and were undergraduates (61.4%).

**Table 1:** Demographic characteristics of the participants.

Variable	Category	Frequency	Percentage (%)
Age (years)	20–29	202	66.7
	30–39	54	17.8
	40–49	23	7.6
	50–59	12	4
	60-69	9	3
Socioeconomic status	Upper class	17	5.6
	Middle class	210	70
	Lower class	73	24.3
Education status	No education	53	17.5
	Undergraduate	186	61.4
	Graduate	45	14.9
	Postgraduate	16	5.3

The sources of breast cancer awareness and their frequencies are shown in Table 2. The most common source of information on breast cancer was friends and family members of the participants.

**Table 2:** Source of information regarding breast cancer.

Source of information	Percent
Friends/Family	45.9
Social media	16.5
School/College	18.5
Awareness programs	13.2
Physicians	5.0

The myths and their prevalence among the participants are shown in Table 3. According to the descriptive analysis, the most common myth was “breastfeeding offers immunity to breast cancer completely” (76.6%), followed by “breast cancer spreads after biopsy” (63.8%).

**Table 3:** Prevalence of myths and misconceptions.

Myth	Prevalence (%)
Is breast cancer contagious?	25.5
Does breast cancer only run in families?	34.3
Only painful lumps are associated with breast cancer?	41.6
Are all lumps in the breast associated with breast cancer?	33.3
Is having breast cancer a license to death?	38.3
Can we treat breast cancer with faith healers and alternative medicine?	47.5
Is breast cancer God’s curse?	31.8
Is breast cancer caused by the evil eye?	38.7
Does breastfeeding prevent cancer 100%?	76.6
Do diagnostic procedures like biopsy cause the spread of cancer?	63.4
Is radiation exposure with mammography responsible for breast cancer?	62.4

The responses of the participants to the myths and misconceptions of the questionnaire are summarized in Table 4.

**Table 4:** Responses of the participants to the myths and misconceptions.

Questions	Yes, N (%)	No, N (%)	I don’t know, N (%)
Do you think every lump in the breast is cancer?	71 (23.4)	199 (65.7)	30 (9.9)
Are only painful lumps associated with breast cancer?	85 (28.1)	174 (57.4)	41 (13.5)
Does breast cancer always run in families?	71 (23.4)	196 (64.7)	33 (10.9)
Is breast cancer God’s curse?	76 (25.1)	205 (67.7)	19 (6.3)
Can the evil eye cause breast cancer?	92 (30.4)	183 (60.4)	25 ( 8.3)
Does breastfeeding offer immunity to breast cancer completely?	165 (54.5)	68 (22.4)	67 (22.1)
Does everyone who has breast cancer die?	81 (26.7)	184 (60.7)	35 (11.6)
Can alternative medicines and faith healers cure breast cancer?	90 (29.7)	156 (51.5)	54 (17.8)
Does breast cancer spread after a biopsy?	116 (38.3)	108 (35.6)	76 (25.1)
Can radiation from a mammogram cause breast cancer?	93 (30.7)	110 (36.3)	97 (32)
Is breast cancer contagious?	55 (18.2)	223 (73.6)	22 (7.3)

The level of education of the participants and the prevalence of their incorrect responses to the myths and misconceptions are shown in Table 5.

**Table 5:** The level of education of the participants and the prevalence of their incorrect responses to the myths and misconceptions.

Questions	Yes, N (%)	No education, N (%)	Primary, N (%)	Secondary, N (%)	Tertiary, N (%)
Do you think every lump in the breast is cancer?	101 (33.3)	34(64.15)	53 (28.49)	10 (22.2)	4 (25.0)
Are only painful lumps associated with breast cancer?	126 (41.6)	38 (71.69)	65 (34.94)	18 (40.0)	5 (31.25)
Does breast cancer always run in families?	104 (34.3)	27 (50.9)	50 (26.8)	18 (40.0)	9 (56.25)
Is breast cancer God’s curse	95 (31.4)	33 (62.26)	46 (24.78)	10 (20.22)	5 (31.25)
Can the evil eye cause breast cancer?	117 (38.7)	35 (66.03)	51 (27.41)	22 (46.88)	9 (56.25)
Does breastfeeding provide 100% immunity against breast cancer?	232 (76.6)	38 (71.6)	149 (80.1)	34 (75.5)	11 (68.7)
Does everyone who has breast cancer die?	116(38.3)	32 (60.3)	60 (32.25)	14 (31.1)	10 (62.5)
Can alternative medicines and faith healers cure breast cancer?	144 (47.5)	28 (52.8)	95 (51.02)	15 (33.3)	6 (32.5)
Does breast cancer spread after a biopsy?	192 (63.4)	40 (75.47)	117 (62.9)	27 (60.0)	8 (50.0)
Can radiation from a mammogram cause breast cancer?	190 (62.7)	39 (73.5)	123 (66.1)	20 (44.4)	8 (50)
Is breast cancer contagious?	77 (25.5)	27 (50.9)	41 (22.04)	7 (15.5)	2 (12.5)

## Discussion

In our study, descriptive statistics were used to determine the proportion of myths and misconceptions regarding breast cancer in our population. These misconceptions were selected after a detailed review of the literature. Pakistan being a Muslim country has various religious beliefs contributing to the misinformation and myths regarding the disease. Cultural values also contribute to the misinformation. According to a systematic review, one factor contributing to the misconception is a health-related concept, which implies that an individual is considered in good health until they can perform their daily life activities without seeking the help of a doctor [12]. This health-related concept is highly prevalent in our population contributing to a significant delay in the diagnosis of breast cancer.

The first misconception analyzed in our study was the fact that participants considered breast cancer to be contagious. According to the results of our study, it was seen that 25.5% of the participants considered breast cancer to be a contagious disease. According to a study conducted by Elobaid et al. in 2016, breast cancer was viewed as a contagious illness contributing to the fear of undergoing screening and being isolated from society [13]. Considering breast cancer as a communicable disease harms the fight against cancer. It was also seen that 34.3% of the participants thought breast cancer ran in families and did not occur in individuals without a relative or family member suffering from breast cancer. A review by van der Groep et al. reported that hereditary breast cancers account for only 5% of the total burden of breast cancers [14]. The most common genes involved in the pathogenesis of breast cancer are the *BRCA1 *and *2*. Therefore, it is imperative to create awareness among the population on the topic of de novo occurrence of breast cancer to guide early detection and management.

Upon determining the presentation of the breast cancer, all participants were asked if all lumps presenting in the breast were considered malignant. One-third (33.3%) of the participants considered all lumps to be breast cancer; however, approximately half (41.6%) of the participants thought that only painful lumps were associated with breast cancer. This finding is in congruence with a study conducted by Altwalbeh et al. in Saudi Arabia in 2015, which also identified that painless breast lumps are perceived as non-malignant and not a threat to life which requires breast cancer screening [15]. This raises a point of concern and requires attention to prevent diagnostic delays in patients with painless breast lumps. In a study by Dye et al. in Ethiopia, the changes in the symptoms associated with breast lumps such as bleeding, increase in size, or pain motivated patients to seek clinical attention [16].

It was determined if participants considered the diagnosis of breast cancer to be a sentence to death. In our study, 38.3% of patients considered that patients who have been diagnosed with breast cancer die and that there is no cure or intervention available for breast cancer. On the other hand, it was also observed that almost half (47.5%) of the participants considered that faith healers and alternative and herbal medicine can provide a cure for breast cancer. The use of holy water for treating conditions such as breast cancer is common in the Muslim population [17]. Cultural and religious taboos are seen to influence the concept of treating breast cancer with faith healers and alternative medications. Widespread cultural beliefs and trust in the power of faith healing also contribute significantly to a delay in diagnosing the disease and presentation to the breast clinics with stage III and IV disease with significant metastasis. This contributes to enhanced morbidity and mortality associated with breast cancer in our population. A delayed stage of presentation also adds to the increased financial burden in the cure, interventions, and palliative care of the patients. In an overview of systematic reviews by Sasaki et al., it has been seen that other factors of alternative measures including a combination of herbal medicine and chemotherapy have shown an improvement in the clinical outcomes of patients [18]. However, in the absence of certified homeopathic doctors and herbal medicine healers, patients are not managed with the right approaches of homeopathic medications, resulting in disastrous effects on the illness and prolonging the time to the first presentation to a healthcare professional.

In our study, 31.4% and 38.7% of the patients thought of breast cancer as God’s curse and a consequence of the evil eye, respectively. In a study by Saeed et al., the most common misconception identified with the causative factor of cancer was fate (82%), followed by 58.8% of participants blaming the evil eye as the cause of their cancer [19]. It is observed that beliefs in myths such as black magic and blaming cancer on God and fate are commonly prevalent in our population. It is important to initiate large-scale efforts to decrease the burden of such myths contributing to breast cancer misinformation and decreased awareness among the masses. Another study among the Omani population by Alkhasawneh et al. also determined that the majority of the participants believed evil eye and envy to be a cause of breast cancer in young women [20]. This belief led to patients considering seeking medical help as futile. Participants considered breast cancer as God’s will and seeking help from healthcare professionals as an interference in God’s plan. Some participants also considered breast cancer as a form of punishment from God or a test that rendered them powerless in seeking any professional help. Participants also felt that the diagnosis of a disease such as breast cancer in women means they have done something bad in the past and it was a form of punishment for their sins.

The most prevalent myth, however, was the prevention of breast cancer with breastfeeding. It was observed that 76.6% of the patients considered that breastfeeding their children would protect them from breast cancer. The idea that breastfeeding offers complete immunity to breast cancer is quite prevalent in the population. It is seen that the duration of breastfeeding does reduce the risk of breast cancer, but is not entirely protective [21]. It is important to promote breastfeeding practices and educate the masses that it reduces the risk of maternal breast cancer; however, is not 100% protective.

Finally, the fear of the spread of breast cancer with biopsy and the use of mammography and radiation exposure for screening purposes was another factor contributing to the delay in management and early detection. More than 60% of the participants believed in the spread of cancer with biopsy and radiation exposure as a causative factor for breast cancer. It was seen that the majority of the misconceptions were more prevalent in the participants with no formal education. In LMICs like Pakistan, the availability of formal education, schools, and colleges is limited. Moreover, it is seen because most of the population falls in the lower socioeconomic group, with the children earning rather than going to schools to receive education. This is another factor contributing to reduced awareness regarding breast cancer in LMICs like Pakistan.

The reasons for the delay in diagnosis include lack of awareness, use of traditional methods, alternative medicine and false belief in the power of faith healers in treating breast cancer, fear of spread with the use of diagnostic modalities, and possible shame and embarrassment. In a study conducted in two hospitals in Pakistan, it was determined that the mean delay in the appearance of a breast lump and first presentation to a primary healthcare physician was 8.79 months [10]. Lower socioeconomic status was one of the major determinants of delayed presentation. Choosing alternative medicine and *Hakeem* for the management of breast cancer was highly linked with a lower education level. Therefore, according to the results of the study, more education and awareness must be created among the masses regarding these misconceptions. This study also suggests utilizing holistic approaches in educating women regarding measures of breast cancer screening and prompt presentation to a healthcare professional to reduce the morbidity and mortality associated with breast cancer.

The limitations of the study include that the data were collected from only one hospital in a metropolitan city of Pakistan; therefore, the data cannot represent the entire Pakistani population. As a result of this limitation, the results of our study may not be generalizable to the entire Pakistani population.

## Conclusions

Healthcare providers should be aware of the myths and misconceptions commonly prevalent in our society. The people of Pakistan are influenced by religious and cultural beliefs associated with a disease such as breast cancer. The low literacy rate of the country adds to the increasing prevalence of such misinformation. This misinformation can be highly persuasive and contributes to diagnostic delay. It is essential to design comprehensive breast cancer education campaigns and awareness programs addressing the myths and misconceptions related to breast cancer to decrease the burden of delayed presentation among Pakistani women.
